# Supplementary figures and images for: Capillary whole-blood IgG-IgM COVID-19 self-test as a serological screening tool for SARS-CoV-2 infection adapted to the general public
Source: PLoS One. 2020 Oct 15;15(10):e0240779. doi: 10.1371/journal.pone.0240779 (PMC7561138; doi:10.1371/journal.pone.0240779)

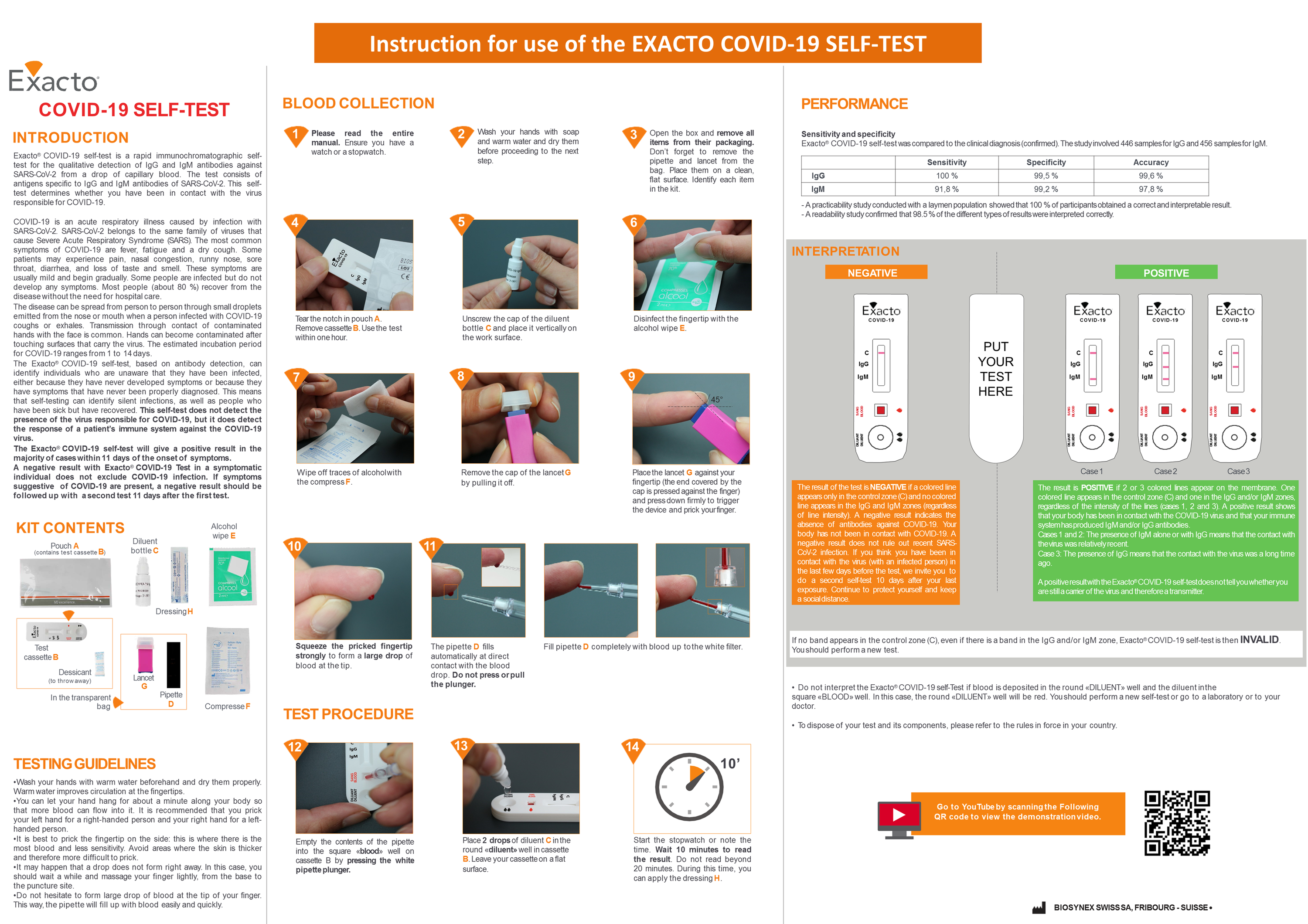

Supplement: S1 Appendix — (TIF) [file pone.0240779.s002.tif]
