# Supplementary material for: Capillary whole-blood IgG-IgM COVID-19 self-test as a serological screening tool for SARS-CoV-2 infection adapted to the general public
Source: PLoS One. 2020 Oct 15;15(10):e0240779. doi: 10.1371/journal.pone.0240779 (PMC7561138; doi:10.1371/journal.pone.0240779)
Supplement: S2 Appendix — (DOCX) [file pone.0240779.s003.docx]

**- Exacto® COVID-19 self-test (Biosynex Swiss SA) -**

**COMPREHENSION DE LA NOTICE**

**Questionnaire d’évaluation de la compréhension de la notice**

***A remplir par le participant.***

Date:…..…/………/………….. addresse:………………………………. ……….....…………………………

Type de notice utilisée: à papier  vidéo

*Après avoir reçu une brève explication des objectifs et du déroulement de l'étude, les participants avaient le choix entre un mode d'emploi sur papier et un mode d'emploi sur vidéo, qu'ils devaient lire ou regarder et comprendre de manière autonome. Après avoir déclaré avoir compris le mode d'emploi, les participants ont été invités à remplir un questionnaire pour évaluer leur compréhension.*

| **Liste des questions liées à la compréhension** | **Réponses du participant** | | |
| --- | --- | --- | --- |
|  | **Vrai** | **Faux** | **Je ne sais pas** |
| Q1 : "Une lettre majuscule est associée à chaque élément du kit pour mieux l'identifier lors de l'exécution de l'auto-test" |  |  |  |
| Q2 : "Le dispositif de prélèvement sanguin (lancette) permet de prélever le sang et de le transférer immédiatement dans le puits SQUARE d'autotest avec la pipette" |  |  |  |
| Q3 : "Deux gouttes de diluant doivent être placées dans le même puits que la goutte de sang". |  |  |  |
| Q4 : "Il faut un chronomètre (montre ou téléphone portable) pour chronométrer 10 minutes avant la lecture du résultat" |  |  |  |
| Q5 : "La présence d'une bande lisible à côté des IgM et/ou des IgG sur la cassette d'autotest signifie que le test est positif". |  |  |  |
| Q6 : "L'absence de résultats de tests par bande est interprétée comme un test négatif". |  |  |  |
| Q7 : "L'absence de bande de contrôle par les résultats des tests doit être interprétée comme un test non valable". |  |  |  |
| Q8 : "Avoir des symptômes moins de 10 jours avant le test ne donne pas un résultat fiable". |  |  |  |
| Q9 : "Si le test est positif, cela signifie qu'ils ont été en contact avec le virus" |  |  |  |
| Q10 : "L'autotest COVID-19 Exacto® ne détecte pas la présence du virus" |  |  |  |

**- Exacto® COVID-19 self-test (Biosynex Swiss SA) -**

**QUESTIONNAIRE DE SATISFACTION EN PHASE PRE-TEST**

***A remplir par le participant.***

|  | | Date : | | | |
| --- | --- | --- | --- | --- | --- |
| Question |  | Facile | Plutôt facile | Plutôt difficile | Très difficile |
|  | *Répondez à cette question en fonction de votre accessoire de sous-étude* |  |  |  |  |
| **1.** | Comment avez-vous trouvé la compréhensibilité des instructions d'utilisation de l'auto-test ? |  |  |  |  |
| **2.** | Comment avez-vous trouvé l'identification des différents composants des kits d'auto-test |  |  |  |  |
| **3.** | Comment avez-vous trouvé la collection d'échantillons ? |  |  |  |  |
| **4.** | Comment avez-vous trouvé le transfert d'échantillons ? |  |  |  |  |
| **5.** | Comment avez-vous trouvé la performance globale de l'auto-test ? |  |  |  |  |
| **6.** | Comment avez-vous trouvé la lecture des bandes après la migration ? |  |  |  |  |
| **7.** | Comment avez-vous trouvé l'interprétation des résultats de l'autotest ? |  |  |  |  |

**- Exacto^®^ COVID-19 self-test (Biosynex Swiss SA) -**

**MANIPULATION DE L’AUTOTEST COVID-19**

**Observation de la manipulation**

*A compléter par l’observateur.*

*L'observateur remet au participant une boîte d'auto-test Exacto® COVID-19 (Biosynex Swiss SA) avec des instructions. L'observateur explique son rôle au participant, et lui précise qu'il jouera le rôle d'un agent de la ligne d'assistance à tout moment pendant le déroulement du test si le participant le demande.*

|  | | DATE:  ……../……../…………. | |
| --- | --- | --- | --- |
| Question |  | **Observation** | **Ask for verbal support** |
|  | *Début d’observation* |  |  |
| **1.** | Le participant a-t-il lu le mode d'emploi ? | **YES**  **/ NO** | **YES**  **/ NO** |
| **2.** | Le participant a-t-il facilement identifié les différents éléments du kit ? | **YES**  **/ NO** | **YES**  **/ NO** |
| **3.** | Le participant s'est-il lavé les mains ? | **YES**  **/ NO** | **YES**  **/ NO** |
| **4.** | Le participant a-t-il correctement retiré la cassette test de la pochette en aluminium ? | **YES**  **/ NO** | **YES**  **/ NO** |
| **5.** | Le participant a-t-il ouvert correctement le flacon de diluant ? | **YES**  **/ NO** | **YES**  **/ NO** |
| **6.** | Le participant a-t-il correctement désinfecté son doigt ? | **YES**  **/ NO** | **YES**  **/ NO** |
| **7.** | Le participant a-t-il essuyé l'alcool résiduel avec la compresse ? | **YES**  **/ NO** | **YES**  **/ NO** |
| **8.** | Le participant a-t-il eu des difficultés à se piquer le doigt ? | **YES**  **/ NO** | **YES**  **/ NO** |
| **9.** | Le participant a-t-il eu des difficultés à former une gouttelette de sang ? | **YES**  **/ NO** | **YES**  **/ NO** |
| **10.** | Le participant a-t-il eu des difficultés à utiliser correctement la pipette jusqu'à ce qu'elle soit remplie jusqu'à la ligne blanche ? | **YES**  **/ NO** | **YES**  **/ NO** |
| **11.** | Le participant a-t-il correctement transféré et déposé le sang dans le puits CARRE de la cassette test ? | **YES**  **/ NO** | **YES**  **/ NO** |
| **12.** | Le participant a-t-il versé deux gouttes de diluant dans le puits ROND de la cassette-test ? | **YES**  **/ NO** | **YES**  **/ NO** |
| **13.** | Le participant a-t-il obtenu un résultat interprétable à la fin du processus malgré une étape manquée ou incorrecte ? | **YES**  **/ NO** | **YES**  **/ NO** |
|  | *Fin d’observation* |  |  |

- **Exacto^®^ COVID-19 self-test (Biosynex Swiss SA) -**

**GRILLE DE LECTURE DES RESULTATS D’UN PANEL D’AUTOTESTS EXACTO® COVID-19 SELF-TEST**

**Interprétation des résultats de l’autotest**

*A compléter par l’observateur.*

*Déroulement de l’étude :*

*1. Vous avez pris connaissance de la grille de lecture de l’autotest à tout moment vous pouvez de nouveau la consulter.*

*2. Parmi les 13 autotests Exacto déjà préparés proposés (ou « cassette »), choisissez en un, au hasard.*

*3. Précisez le numéro du test sur cette fiche.*

*4. Demandez et notez le résultat lu par le participant, et notez le résultat attendu.*

*5. Répétez les étapes 3 et 4 pour 12 autres tests que vous tirerez au hasard parmi les tests restants.*

| **Test number**  **Cassette number** | **Résultats lus par le participant** | | | | **Résultat lu par l’observateur** | | |
| --- | --- | --- | --- | --- | --- | --- | --- |
|  | **POSITIF** | **NEGATIF** | **INVALIDE** | **JE NE SAIS PAS PASOW** | **Positif** | **Négatif** | **Invalide** |
|  |  |  |  |  |  |  |  |
|  |  |  |  |  |  |  |  |
|  |  |  |  |  |  |  |  |
|  |  |  |  |  |  |  |  |

**- Exacto^®^ HIV Test -**

**QUESTIONNAIRE DE SATISFACTION**

**Satisfaction concernant la réalisation et la lecture de l’autotest**

*A compléter par le participant.*

**Votre profil :**

- **Age :** …………….. ans
- **Si femme, êtes-vous enceinte avec test de grossesse positif ?** Oui  Non
- **Etat civil :** Célibataire  Mariage ou union libre  Veuf(ve)  Divorcé(e) ou séparé(e)
- **Occupation :** Elève/Etudiant  Avec emploi  Sans emploi
- **Niveau d’étude :** Non scolarisé  Primaire  Secondaire

Supérieur ou universitaire : 1^er^ cycle  2^e^ cycle  3^e^ cycle

- **Aviez-vous eu des symptômes de la COVIS-19 ces deux dernier mois ?** Oui  Non
- **Aviez-vous bénéficié d’un test moléculaire utilisant après écouvillonnage naso-pharyngien ?**

Oui  Non

- **Si oui, le résultat était-il positif ?** Yes  No

**Questionnaire de satisfaction post-test**

|  | | Date : | | | |
| --- | --- | --- | --- | --- | --- |
| Question |  | Facile | Plutôt facile | Plutôt difficile | Très difficile |
|  | *Répondez à cette question en fonction de votre accessoire de sous-étude* |  |  |  |  |
| **1.** | Comment avez-vous trouvé la compréhensibilité des instructions d'utilisation de l'auto-test ? |  |  |  |  |
| **2.** | Comment avez-vous trouvé l'identification des différents composants des kits d'auto-test |  |  |  |  |
| **3.** | Comment avez-vous trouvé la collection d'échantillons ? |  |  |  |  |
| **4.** | Comment avez-vous trouvé le transfert d'échantillons ? |  |  |  |  |
| **5.** | Comment avez-vous trouvé la performance globale de l'auto-test ? |  |  |  |  |
| **6.** | Comment avez-vous trouvé la lecture des bandes après la migration ? |  |  |  |  |
| **7.** | Comment avez-vous trouvé l'interprétation des résultats de l'autotest ? |  |  |  |  |
| **8.** | Aviez-vous facilement surmonté les difficultés rencontrées ? |  |  |  |  |
