# Supplementary material for: Capillary whole-blood IgG-IgM COVID-19 self-test as a serological screening tool for SARS-CoV-2 infection adapted to the general public
Source: PLoS One. 2020 Oct 15;15(10):e0240779. doi: 10.1371/journal.pone.0240779 (PMC7561138; doi:10.1371/journal.pone.0240779)
Supplement: S3 Appendix — (DOCX) [file pone.0240779.s004.docx]

**- Exacto® COVID-19 self-test (Biosynex Swiss SA) -**

**UNDERSTANDING OF THE INSTRUCTIONS**

**Questionnaire to assess understanding of the instructions**

***To be completed by the participant.***

Date:…..…/………/………….. address:………………………………. ……….....…………………………

Type of instruction for use: paper-based  video-based

*After receiving a brief explanation of the objectives and conduct of the study, the participants had the choice between a paper-based instruction for use and a video-based instruction for use, which they were asked to read or watch and understand independently. After their self-declaration of having understood the instruction for use, the participants were asked to fill a questionnaire to gauge their comprehension.*

| **Comprehension of labeling checklist^*^** | **Participants' responses** | | |
| --- | --- | --- | --- |
|  | **True** | **False** | **Don’t know** |
| **Q1**: “A capital letter is associated with each component of the kit to better identify it during the performance of self-test” |  |  |  |
| **Q2**: “The blood collection device (lancet) helps to collect the blood and transfer it immediately into the SQUARE well of self-test with the pipette” |  |  |  |
| **Q3**: “Two drops of diluent should be placed in the same well as the drop of blood” |  |  |  |
| **Q4**: “A timer (watch or mobile) to clock 10 minutes before reading the result is need” |  |  |  |
| **Q5**: “Presence of a readable strip next to IgM and/or IgG on the self-test cassette means that the test is positive” |  |  |  |
| **Q6**: “Lack of band by test results is interpreted as a negative test” |  |  |  |
| **Q7**: “Lack of control band by test results should be interpreted as an invalid test” |  |  |  |
| **Q8**: “Having symptoms less than 10 days before the test does not provide a reliable result” |  |  |  |
| **Q9**: “If the test is positive it means that they have been in contact with the virus” |  |  |  |
| **Q10**: “The Exacto® COVID-19 self-test does not detect the presence of the virus” |  |  |  |

**- Exacto® COVID-19 self-test (Biosynex Swiss SA) -**

**pre-TEST SATISFACTION QUESTIONNAIRE**

*To be completed by the participant*

|  | | Date: | | | |
| --- | --- | --- | --- | --- | --- |
| Items |  | Easy | Rather easy | Rather difficult | Difficult |
|  | *Complete this question according your substudy appurtenance* |  |  |  |  |
| **1.** | How did you find the understandability of instructions for use of self-test? |  |  |  |  |
| **2.** | How did you find the identification of the different components of the self-test kits |  |  |  |  |
| **3.** | How did you find the sample collection? |  |  |  |  |
| **4.** | How did you find the sample transfer? |  |  |  |  |
| **5.** | How did you find the overall performance of self-test? |  |  |  |  |
| **6.** | How did you find the reading of strips after migration? |  |  |  |  |
| **7.** | How did you find the interpretation of self-test results? |  |  |  |  |

**- Exacto^®^ COVID-19 self-test (Biosynex Swiss SA) -**

**handling of the self-test by a LAY user**

**Observation of the handling of the self-test by a lay user**

*To be completed by the observer.*

*The observer gives the participant an Exacto^®^ COVID-19 self-test (Biosynex Swiss SA) box with instructions. The observer explains his/her role to the participant, and explains that he/she will play the role of a helpline agent at any time during the handling of the test if the participant requests it.*

|  | | DATE:  ……../……../…………. | |
| --- | --- | --- | --- |
| Items |  | **Observation** | **Ask for verbal support** |
|  | *Start time of observation* |  |  |
| **1.** | Did the participant read the instruction for use? | **YES**  **/ NO** | **YES**  **/ NO** |
| **2.** | Did the participant easily identify the different components of the kit? | **YES**  **/ NO** | **YES**  **/ NO** |
| **3.** | Did the participant wash his hands? | **YES**  **/ NO** | **YES**  **/ NO** |
| **4.** | Did the participant properly remove the test cassette from the aluminum pouch? | **YES**  **/ NO** | **YES**  **/ NO** |
| **5.** | Did the participant open the diluent vial correctly? | **YES**  **/ NO** | **YES**  **/ NO** |
| **6.** | Did the participant disinfect his finger correctly? | **YES**  **/ NO** | **YES**  **/ NO** |
| **7.** | Did the participant wipe residual alcohol with the compress? | **YES**  **/ NO** | **YES**  **/ NO** |
| **8.** | Did the participant have difficulty lancing their finger? | **YES**  **/ NO** | **YES**  **/ NO** |
| **9.** | Did the participant have difficulty forming a blood droplet? | **YES**  **/ NO** | **YES**  **/ NO** |
| **10.** | Did the participant have difficulty using the pipette correctly until it was filled up to the blank line? | **YES**  **/ NO** | **YES**  **/ NO** |
| **11.** | Did the participant correctly transfer and deposit the blood into the SQUARE well of the test cassette? | **YES**  **/ NO** | **YES**  **/ NO** |
| **12.** | Did the participant shed two drops of diluent in the ROUND well of the test cassette? | **YES**  **/ NO** | **YES**  **/ NO** |
| **13.** | Did the Participant obtain an interpretable result at the end of the process despite a missed or incorrect step? | **YES**  **/ NO** | **YES**  **/ NO** |
|  | *End time of handling* |  |  |

- **Exacto^®^ COVID-19 self-test (Biosynex Swiss SA) -**

**READING GRID FOR THE RESULTS OF PRE-PREPARED SAMPLES OF THE EXACTO® COVID-19 SELF-TEST**

**Interpretation of the results of the self-test**

*To be completed by the observer.*

*Running the study:*

*1. You have read the reading grid for the Exacto® COVID-19 self-test. You can consult it again at any time.*

*2. From the 8 pre-prepared Exacto® COVID-19 self-test proposed (or “cassette”), choose one at random.*

*3. Write down the number of the test on this form.*

*4. Ask for and write down the result read by the participant, and write down the expected result.*

*5. Repeat steps 3 and 4 for 3 other tests that you will choose at random from the remaining tests.*

| **Test number**  **Cassette number** | **Result read by the volunteer** | | | | **Expected result** | | |
| --- | --- | --- | --- | --- | --- | --- | --- |
|  | **POSITIVE** | **NEGATIVE** | **INVALID** | **DOESN'T KNOW** | **Positive** | **Negative** | **Invalid** |
|  |  |  |  |  |  |  |  |
|  |  |  |  |  |  |  |  |
|  |  |  |  |  |  |  |  |
|  |  |  |  |  |  |  |  |

**- Exacto^®^ HIV Test -**

**POST-TEST SATISFACTION QUESTIONNAIRE**

*To be completed by the participant.*

**Your profile:**

- **Age:** …………….. years
- **Sex:** Female  Male
- **If you are a woman, are you pregnant with a positive pregnancy test?** Yes  No
- **Marital status:** Single  Married or cohabiting  Widowed  Divorced or separated
- **Occupation:** Student  Employed  Unemployed
- **Level of education:** Unschooled  Primary  Secondary

Higher or university: Bachelor  Master  Doctorate

- **Did you have the symptoms of COVID-19 in the past two months?** Yes  No
- **Have you previously tested for COVID-19 molecular testing (nasopharyngeal swab)?**

Yes  No

- **If yes, did you have a positive result for COVID-19?** Yes  No

**Satisfaction questionnaire**

|  | |  |  |  |  |
| --- | --- | --- | --- | --- | --- |
| Items |  | Easy | Rather easy | Rather difficult | Difficult |
|  | *Complete this question according your substudy appurtenance* |  |  |  |  |
| **1.** | How did you find the understandability of instructions for use of self-test? |  |  |  |  |
| **2.** | How did you find the identification of the different components of the self-test kits |  |  |  |  |
| **3.** | How did you find the sample collection? |  |  |  |  |
| **4.** | How did you find the sample transfer? |  |  |  |  |
| **5.** | How did you find the overall performance of self-test? |  |  |  |  |
| **6.** | How did you find the reading of strips after migration? |  |  |  |  |
| **7.** | How did you find the interpretation of self-test results? |  |  |  |  |
| **8.** | How did you find your ability to surmount the difficulties encountered? |  |  |  |  |
