# Supplementary material for: Capillary whole-blood IgG-IgM COVID-19 self-test as a serological screening tool for SARS-CoV-2 infection adapted to the general public
Source: PLoS One. 2020 Oct 15;15(10):e0240779. doi: 10.1371/journal.pone.0240779 (PMC7561138; doi:10.1371/journal.pone.0240779)
Supplement: Attachment — Submitted filename: Responsestoreviewers29092020lb.docx [file pone.0240779.s008.docx]

**Responses to journal requirements and to Reviewers**

***Journal Requirements:***

***Reviewers' comments:***  *Reviewer's Responses to Questions*

***Comments to the Author*** *1. If the authors have adequately addressed your comments raised in a previous round of review and you feel that this manuscript is now acceptable for publication, you may indicate that here to bypass the “Comments to the Author” section, enter your conflict of interest statement in the “Confidential to Editor” section, and submit your "Accept" recommendation.*

*Reviewer #1: (No Response)*

*Reviewer #2: All comments have been addressed*

**Our answer:** We have made minor corrections thorough the manuscript; therefore, we hope that our revised manuscript is presented in an intelligible fashion and written in standard English.

*2. Is the manuscript technically sound, and do the data support the conclusions?*

*The manuscript must describe a technically sound piece of scientific research with data that supports the conclusions. Experiments must have been conducted rigorously, with appropriate controls, replication, and sample sizes. The conclusions must be drawn appropriately based on the data presented.*

*Reviewer #1: Yes*

*Reviewer #2: (No Response)*

*3. Has the statistical analysis been performed appropriately and rigorously?*

*Reviewer #1: Yes*

*Reviewer #2: (No Response)*

*4. Have the authors made all data underlying the findings in their manuscript fully available?*

*The PLOS Data policy requires authors to make all data underlying the findings described in their manuscript fully available without restriction, with rare exception (please refer to the Data Availability Statement in the manuscript PDF file). The data should be provided as part of the manuscript or its supporting information, or deposited to a public repository. For example, in addition to summary statistics, the data points behind means, medians and variance measures should be available. If there are restrictions on publicly sharing data—e.g. participant privacy or use of data from a third party—those must be specified.*

*Reviewer #1: Yes*

*Reviewer #2: Yes*

**Our answer:** We thank the reviewers for their nice comments on our work.

*3. Have the authors made all data underlying the findings in their manuscript fully available?*

*The PLOS Data policy requires authors to make all data underlying the findings described in their manuscript fully available without restriction, with rare exception (please refer to the Data Availability Statement in the manuscript PDF file). The data should be provided as part of the manuscript or its supporting information, or deposited to a public repository. For example, in addition to summary statistics, the data points behind means, medians and variance measures should be available. If there are restrictions on publicly sharing data—e.g. participant privacy or use of data from a third party—those must be specified.*

*Reviewer #1: Yes*

*Reviewer #2: Yes*

**Our answer:** We thank the reviewers for their nice comments on our work.

*5. Is the manuscript presented in an intelligible fashion and written in standard English?

PLOS ONE does not copyedit accepted manuscripts, so the language in submitted articles must be clear, correct, and unambiguous. Any typographical or grammatical errors should be corrected at revision, so please note any specific errors here.*

*Reviewer #1: No*

*Reviewer #2: Yes*

**Our answer:** In order to acknowledge the comments raised by Reviewer # 1, we have corrected words and grammar as suggested by Referee.

*5. Review Comments to the Author*

*Please use the space provided to explain your answers to the questions above. You may also include additional comments for the author, including concerns about dual publication, research ethics, or publication ethics. (Please upload your review as an attachment if it exceeds 20,000 characters)*

***Answer to reviewer #1***

*Further minor corrections are required. There are still language problems.*

**Our answer:** In order to acknowledge the comments raised by Reviewer # 1, we have corrected all the confusing sentences raised by the referee.

*Note [page 18]: L74: please clarify what ‘overcoming the de-confinement period’ means*

*Highlight [page 18]: Furthermore, antibody tests for SARS-CoV-2 may constitute one of the keys to fight the SARS-CoV-2 epidemic, in particular to overcome the de-confinement period [9].*

**Our answer:** We have changed the sentence as following: “*Furthermore, antibody tests for SARS-CoV-2 may constitute one of the keys to fight the SARS-CoV-2 epidemic, in particular to overcome the period after lockdown”.*

*Note [page 18]: L76 Delete a priori considered to be healed and protected against new infection*

*Highlight [page 18]: Seropositivity to SARS-CoV-2 antigens would also allow to identify previously infected individuals, including asymptomatic patients, a priori considered to be healed and protected against new reinfection [9].*

**Our answer:** We have simplified this sentence as suggested.

*Note [page 21]: L140 change is to was*

**Our answer:** We have changed the words as suggested.

*Note [page 26]: L270- insert (Q10) after question*

**Our answer:** Done

*Note [page 26]: Table 1; Previously diagnosed COVID-19 positive among those previously COVID-19 tested. Given the total number is 22+145= 167 shouldn’t the ‘among those previously COVID-19 tested be removed? The previous line of the table indicates only 34 were tested.*

**Our answer:** We thank the reviewer for this pertinent remark. This is a mistake. Indeed, of the 34 people who were previously tested, 22 were found to be positive for SARS-CoV-2 infection. We have corrected this in the revised manuscript.

*Note [page 28]: L296: change 'oriented' to 'referred to a clinically certified laboratory'*

*Highlight [page 28]: Note that, in this substudy, 11 (13.3%) people had a positive results with the self-test, and they were oriented to laboratory for result confirmation.*

**Our answer:** We have changed the words as suggested.

*Note [page 32]: L380 to 384: better to break this long sentence down into at least two smaller and easier to understand sentences.*

**Our answer:** We have changed the sentence as suggested.

*Note [page 35]: L 447 change 'pseudo-insurance of immunity or non-contagiousness' to ‘an individual assuming they are immune or non-contagious when they are not. This emphasizes the need for pre- and post-test counseling.’*

*Highlight [page 35]: The possible risk of adverse effects of the COVID-19 self-test should not be underestimated, such as a pseudo-insurance of immunity or non-contagiousness.*

**Our answer:** We have changed the sentence as suggested.

*Highlight [page 35]: It will of course be necessary to precisely assess all these potential perverse effects.*

*Note [page 35]: L450 change 'However,' to 'Nevertheless,'*

*Note [page 35]: L 449 change sentence’ It will of course..” to “Post-marketing surveillance for these potential adverse consequences will be needed.”*

**Our answer:** We have changed the sentence as suggested.

*Note [page 36]: L456 delete full*

**Our answer:** We have deleted the word as suggested.

*Note [page 37]: l481 change ‘does not eliminate a SARS-CoV-2 infection’ to ‘does not exclude the possibility of active SARS-CoV-2 infection and infectiousness’’*

**Our answer:** We have changed the sentence as suggested.

*Note [page 37]: L484 change’ accentuating tensions in’ to ‘a burden on’*

**Our answer:** We have changed the sentence as suggested.

*Note [page 37]: L 485. Change ‘ In any case, the presence of ….”immunological passport” of protection ….immunity (49)’ to “It should be emphasized that it is not known if a positive antibody test represents protection and the concept of an “immunological passport” cannot be supported at this time. (46,47,49)” This change was previously requested and is essential.*

*Highlight [page 37]: In any case, the presence of reactivities could constitute an "immunological passport" of protection [46,47], although the general assumption is that the presence of antibodies will provide at least some immunity [49].*

**Our answer:** We have changed the sentence as suggested.

*Note [page 37]: L487: change 'However,…' sentence to “ Even if antibodies to SARS-CoV-2 are shown to offer some level of protection the durability of any such protection is not known at this time. Furthermore the presence of antibodies does not necessarily indicate that the person is not contagious particularly during the early phases of infection ”*

**Our answer:** We have changed the sentence as suggested.

*Note [page 37]: L500 Change ‘will’ to ‘may’*

**Our answer:** Done

*Note [page 37]: L 503: delete ‘,lead to partial lifting of “stay-at-home” or “shelter-in-place” orders, and would help get the economy back to normal’*

**Our answer:** We have deleted the sentence as suggested.

*Note [page 38]: L 505 change to ‘UK, has made SARS-CoV-2 home tests available to healthcare workers… ‘*

**Our answer:** We have changed the sentence as suggested.

*Note [page 38]: L 510: delete’ to individuals and the country’*

**Our answer:** We have deleted the sentence as suggested.

*Note [page 38]: L 510 : change ‘features demonstrate’ to ‘study demonstrates’*

**Our answer:** We have changed the words as suggested.
